# Buffalo Pathological Society

**Published:** 1891-04

**Authors:** 


### BUFFALO PATHOLOGICAL SOCIETY.

STATED MEETING MARCH 13, 1891.

The President, Dr. Charles G. Stockton in the chair.

Dr. Frank H. Potter, Secretary *pro tem.*

Dr. Peter C. Cornell applied for membership and presented the following report with specimens:

Captain Mc——; age about fifty. Health had been apparently good up to within a few weeks of his visit at the office, about the middle of last September, when he complained of being generally run down; of some pain in upper part of back between the shoulder blades, and a cough. It was considered that he was run down from hard work, and as examination of chest indicated only sub-acute bronchitis and a double aortic murmur, he was not thought to be seriously ill; a plaster and stimulating expectorant were ordered with advice to lay off a trip and recuperate. He continued at his work, but on his return two weeks later took to his bed. A marked change had now taken place and he had the appearance of being a very sick man; complained of shortness of breath; cough, with moderate expectoration of frothy mucus, and at intervals a slight quantity of pure blood. The pain between the shoulder blades, which had at first been slight, was now very severe and there was great prostration. A careful examination at this time showed congestion of both lungs and a tumultuous heart.

While the man was desperately sick, the diagnosis of the exact cause of his illness was not altogether clear, his serious condition not being entirely accounted for by physical signs. The expectoration of pure blood suggested the existence of an aneurism; but, inasmuch as until within two weeks of his death he had been an apparently healthy lake captain in active service, and the examination of his chest revealed no aneurismal sign, it was only considered as possible. Under the use of opiates he passed a fairly good night, his condition, however, showing no improvement; the expectoration of pure blood continuing with a a little more frequency. This condition of affairs continued with a gradual growing worse until after midnight, when he was seized with a sudden gush of blood from his mouth, and died almost instantly.

Examination of the thorax, thirty hours after death, showed a moderate amount of pleuritic adhesions, evidence of bronchitis, and congestion of both lungs. Upon passing the hand posteriorly a large aneurism of the aorta was discovered extending from the fifth to the tenth dorsal vertebra, the bodies of which were almost destroyed by erosion; the remaining partition between the spinal canal being so thin as to crumble away during the examination.

In presenting this clinical memorandum and specimen, the following points are of interest:

*First.*—That such extensive damage to the principal blood-vessel and to the vertebræ should exist, and at the same time permit the man to continue at his laborious work.

*Second.*—That the physical signs and clinical evidence of aneurism, particularly such an extensive one, should be unappreciable.

Upon ballot Dr. Cornell was declared elected to membership.

Dr. B. C. Johnson then read, by invitation, the clinical history and reported the post-mortem following upon a case in Dr. Stockton's ward at the General Hospital :

HISTORY OF A CASE OCCURRING AT THE BUFFALO GENERAL HOSPITAL.

Patsy S., aged thirteen; born in United States of Irish descent. Father living and healthy. Mother said to have died of pneumonia, after an illness of six weeks. Five brothers and five sisters. Of the brothers, two were of still birth and two died of cholera infantum. Two sisters dead, at the ages of one and three years respectively. Three sisters living and in apparently good health. Patient's occupation was that of a signalman, over hatchways upon canal boats, and had prior to his last illness enjoyed remarkably good health.

He was strong, well nourished, rugged and hearty. He had at times been in the habit of imbibing quite freely, somtimes even to intoxication, and was frequently exposed to inclemencies of weather, as by lying out in open air all night during the Fall and Winter months, averaging twice weekly. During early childhood patient was of unusually good health, having none of the eruptive diseases, save measles at the age of three.

Three months prior to entering the hospital he first began to be troubled with slight attacks of vertigo, lasting for a few minutes at a time, and occurring twice weekly for one month, when in addition to these he began to have pain in the temporal region, sharp and cutting in character.

He stopped work at this time and attempted to attend school, but the attacks occurring more frequent and blinding him for the time being, he was obliged to leave. He continued to have these attacks of about the same character until December 1st, three days prior to entering hospital, when they suddenly became of greater severity and at shorter intervals, occurring three times daily and lasting from fifteen to twenty minutes each time. They would commence, as did the others, with severe pain in the temporal region, head drawn backwards to a marked degree, becoming dizzy and he would fall were he not supported, yet losing consciousness none of the time. At the expiration of the attack he would feel no different than at any time. Appetite good but easily nauseated and vomiting soon as food entered his stomach. Upon entering the hospital his head was extended upon his shoulders with considerable rigidity, and upon flexing his head he would cry out with pain. Pupils symmetrical, yet somewhat dilated. Temperature normal, or not ranging above 99° F. He would lie in a semi-comatose condition, yet easily awakened when aroused, would complain of severe pain extending to the occipital region and constantly remaining in the temporal and frontal regions, with exacerbations of a more intense character. He would lie perfectly quiet until these attacks came on, which at this time was about hourly.

At 1 P. M., December 5th, twenty-four hours after entering, during one of these attacks, not dissimilar to the preceding ones, with no premonitory signs or symptoms, no complaint of pain, no convulsion, respiration suddenly ceased, he became greatly cyanosed, muscles relaxed and pupils widely dilated. His heart was found to be beating and regular although feebly, and pulse not perceptible at the wrist. Sylvester's method of artificial respiration was commenced. It was found by the vigorous use of this method that the cyanosis was relieved, and the pulse reappeared at the wrists, yet upon withholding the artificial measures respiration ceased and never after was one voluntary effort at respiration noticed. In hopes that voluntary respiration would after a time commence, this method was kept up until 6 P. M., the heart remaining as strong in its beat, the patient making no move during the entire time, and upon withholding the artificial respiration cyanosis appearing and heart failing and patient becoming pulseless.

At 6 P. M., tracheotomy was performed by Dr. Stockton, and by means of a crude bellows with a soft flexible catheter attached to a tracheotomy tube, mechanical respiration was carried on.

During this time he had been given rectal enemata of warm coffee and brandy, hypodermic injections of tr. digitalis, and glonoin in ten and four drop doses respectively at two different times, also 1-60 grain of strychnia sulph., hypodermically.

At 11 P. M., movement was for the first time noticed, and again thirty minutes later slight flexion of both lower extremities occurred. From this time on he continued in about the same condition with incontinence of urine and making no voluntary motion until 1 A. M., when his heart becoming suddenly weaker he at last expired, exactly twelve hours after artificial respiration was commenced.

Autopsy, December 6th, fifteen hours after death, made by Dr. Bergtold. Body well nourished and showed several small bruises and excoriations upon the arms, (the result of artificial respiration).

*Brain.* – The superior longitudinal sinus was engorged with blood, also the dural vessels. Cortex remarkably dry and convolutions flattened. Vessels of cortex and pia mater were markedly injected. The pia mater over many sulci was opaque, white and thickened. No pus or milliary tubercles visible. Frontal lobes were mutually adherent by their internal surfaces. Pia at the base of the brain was somewhat thickened and opaque. All the ventricles were greatly dilated, fluid oozing up as soon as lateral ventricles were opened which were found to contain two ounces apiece of cloudy fluid. No fresh lymph visible anywhere. All of the tissues adjacent to the lateral, third and fourth ventricles, were softened, wet and almost diffluent. This includes the surfaces of the corpora striata, optic thalami, ependema, corpus collosum, etc. From the floor of the fourth ventricle to the left side, there projected a small gobular mass about the size of a split pea, which was fluctuating. Incision into it allowed considerable semi-solid granular matter to escape. The softening had also attacked the medulla, the interior of which was filled with semi-solid *débris*, while upon the outside gave a sense of fluctuation. Vessels at the base were a trifle more opaque than normal. No milliary tubercles seen along their course and no infarcts found. No thrombi found in any of the vessels of the brain, although careful examination was made for them. Weight of brain forty-six and one-half ounces.

*Thorax.*—Pluræ normal. Lungs normal, save a slight amount of congestion at each base. Slight amount of emphysema, and in the apex of the right lung was found a circumscribed mass of caseous material about as large as a chestnut; also, one of the right bronchial nodes had become caseated.

*Heart*, normal, save a slight amount of atheroma in very small spots in the beginning of the aorta, and an apparent thickening of the mitral valves, and upon one of its leaflets were three small bright-red petechiæ. No ulcerations. Heart full of dark, thick blood. Weight, eight and one-half ounces.

*Spleen, Kidneys*, pancreas, and intestines, normal. Mesenteric lymph nodes enlarged. Stomach slightly dilated; walls thickened, and inner surface covered by thick, white mucus.

*Liver*, normal.

The paper of the evening was then read by Dr. W. H. Bergtold.

### HODGKIN'S AND ALLIED DISEASES.

During the year 1889 there were reported, according to Sajous' Annual, in the various medical journals of the world, only five cases of Hodgkin's disease. The following is, therefore, reported principally because of this infrequency, and also hoping that it may lead to a brief discussion on its pathology, and to a comparison of the interesting points of relation of this malady to another closely allied.

Case.—J. C., aged 27; family history unimportant, always healthy until one year previous to entrance into hospital, at which time the lymphatics at the angle of the jaw slowly enlarged, the growth gradually involving the cervical lymphatic chain down to the clavical, both sides being affected, the left later; patient felt weak and tired on exertion. Condition at entrance: Cervical lymphatics on both sides from angle of jaw to clavicle greatly enlarged, freely movable under skin and not tender; thyroid, slightly enlarged; axillary and inguinal glands also increased in size. No noticeable splenic tumor ; no tenderness over bony surfaces or points. Is breathless on exertion. No record of urinary examination. From entrance until death, or about one and one-half years after the beginning of the disease, there was a steady progression of the symptoms; the thyroid enlarged so much that shortly before death it so compressed the trachea that a tracheotomy was made to save the patient from immediate suffocation. About this time the right elbow began to enlarge, and became painful. Twenty days before death a count of the blood cells gave a result as follows: reds, 2,768,750 ; whites, 18,750 per cubic mm., a condition of leucocytosis not sufficient to be called leucocythemia. The normal number of reds being about 5,000,000 per c.mm., and of whites about 10,000 ; the reds thus here being reduced about fifty per cent. and the whites increased in about the same proportion. Death occurred from exhaustion. A few weeks before this event, Dr. Williams, the house surgeon, removed a small inguinal lymph node under cocaine and kindly gave it to me for study. An examination enabled me to make a diagnosis of at least lymphoma, with a tentative one of Hodgkin's disease, a blood count at that time excluding leukemia. Autopsy resulted as follows :

*Post-mortem* on body of male, apparently about thirty years old. High degree of emaciation ; rigor mortis not present ; ends of the bones entering into formation of right elbow markedly enlarged ; neck presented the closed wound of a recent tracheotomy ; cervical, axillary, and inguinal lymphatics markedly enlarged. Arch of diaphragm on both sides rose to the fifth rib. Right plura adherent at apex, otherwise normal, left also adherent at apex ; cavity contained about 100 c.c. of slightly bloody serum. The pericardium contained 50 c.c. of clear serum, otherwise normal. The heart was very small, weighing about 210 gm.; myocardium seemed normal ; right and left endocardium, and all the valves were normal ; commencement of the aorta presented a few patches of atheroma, not calcarious ; left ventricular cavity very small, and whole heart empty. Lymph nodes at lower end of mediastinum enlarged to a diameter of about 1.5 cm. Lungs : right apex adherent ; adhesions old and firm ; firm interlobular adhesions ; no tubercles or nodules. Left apex adherent, old and firm ; no tubercles or nodules ; slight amount of purulent matter expressible from smaller bronchi of right lung ; bronchial lymph nodes enlarged to a diameter of 2 cm. pigmented in centre, and periphery of a pale yellow color, firm in consistency, and not succulent; left bronchial lymph nodes same as right, but three, however, presenting caseous centers. Post mediastinal lymphatics greatly enlarged, some to 4 cm. in diameter, whitish yellow on section, firm, and not caseated or pigmented. Each lobe of the thyroid as big as a walnut (4 cm. in diameter). Spleen normal in size, slightly adherent at upper edge ; capsule not thickened ; substance on section presented round, whitish-yellow masses about 1 cm. in diameter, rather firm, similar in appearance to the enlarged nodes found elsewhere. Kidneys: Left, normal in size, blue in color, firm, capsule adherent, cortex narrowed, no nodules. Right, contracted pale and firm; capsule, adherent; organ contained no nodules. Adrenals were each enlarged, right more than the left, it being about 6.25 cm. long, 2.50 cm. broad and 1 cm. thick. Each contained nodules similar in texture and appearance to those of the spleen, but smaller. The intestines were injected, mesenteric lymph nodes were markedly enlarged; ascending colon exhibited several nodules on the surface of the peritoneum opposite the mesenteric attachment; these nodules were movable. In the ileum several Peyer's patches were markedly thickened, and on section gave a similar appearance as the nodules of the spleen. The retroperitoneal lymphatics were greatly enlarged. The stomach, pancreas, and liver seemed normal. The brain was not examined. All of the lymph nodes, the retro-peritoneal, post-mediastinal, axillary, mesenteric, etc., gave a like picture on section: namely, whitish-yellow in color, firm in consistence, not juicy and not caseated.

Microscopic examination of these various tissues gave results more or less in accord with the description of the lesions usually found in pseudo-leucocythemia. The marrow of the bones (sternum) seemed about normal, though if there was any change it may be said to be only a slight increase of the lymphoid elements. In the liver, there was a slight increase of connective tissue about the vessels and in the interlobular spaces, with, however, no new deposits of lymphoid cells. The liver, otherwise, was normal. The kidneys had some of the tubules replaced by connective tissue; much of the tubular epithelium was in a state of parenchymatous degeneration, and the capsule was thickened. The lung presented numerous areas of lymphoid tissue, each being placed near a bronchus; there was also marked emphysema and chronic interstitial pneumonia.

In the spleen was found a marked increase of trebecular tissue, and a complete diffusion of lymphoid cells; mixed with the usual spleen pulp and blood débris. The ordinary structure of Malpighian bodies, lymph cords, etc., was lost in this dissemination of new cells. There were also great areas which took stains poorly, and which presented the characteristics of coagulation necrosis. The lymph-nodes all showed marked increased connective tissue stroma, and the ordinary arrangement of follicle, perifollicular space, etc., was destroyed, the lymphoid elements having disseminated themselves throughout each node.

Three bronchial nodes were caseous. All of these tissues were stained for microörganisms, both by the Koch-Ehrlich method for the bacillus of tuberculosis, and by Gram's method for the usual other bacteria ; in all of these stainings not a single germ was found, though I doubt not had the caseous nodes been searched closely enough, the presence of the bacillus of tuberculosis would have been found.

An examination of the names given to this malady at various times would enable one to gather a fairly good idea of it as a disease. Thus, from Trousseau's name, "adenia," one would be led to infer that there was an involvement of the glands, though we would not know whether a true secretory gland, as the parotid, was implied, or a so-called lymphatic gland. This is just as good a time as any to stop and call attention to the fact that the name commonly given to those collections of lymphatic tissue, known as lymphatic glands, is a misnomer, and that they are not glands at all, having no true glandular structure, and no excretory product or duct. Such collections are more properly termed "lymph-nodes." Wilk's name, lymphatic anemia, leads one to learn that the disease expresses itself in one direction as an anemia and of lymphatic origin, and necessarily severe because prominent enough to characterize the malady in naming it. This name is open to objection, however, for we have anemias with the so-called scrofulous enlargement of the lymphatics. But it is proper and well-chosen on the score that it reminds us that the anemia is secondary to the lymphatic changes, a point of much importance, inasmuch as it is not at all rare to find a certain degree of enlargement of the lymph nodes in anemia of the so-called primary origin.

Hodgkin's disease is also known by a number of other names, some of which may be incidentally mentioned farther on. That one given to it by Langhans is perhaps the best, he calling it "pseudo-leukemia lymphomatosis"; the name adopted as the title of this paper is chosen solely because of its being the most familiar to English readers. No brief definition can be applied to Hodgkin's disease. It is characterised by a progressive enlargement of the lymph nodes throughout a large extent of the body, or in the whole of the body, at times slowly and then again rapidly affecting different parts ; with this increase in size there is no accompanying tendency to inflammation, suppuration, caseation, or confluency ; there may or may not be enlargement of the spleen ; there usually is great and marked anemia, and rarely a sufficient degree of leucocytosis to admit of leucocythemia.

The disease seems to partake of an infectious nature, for the lymph nodes do not necessarily become involved by contiguity, but, on the contrary, the second locality may and often is quite remote from the primary manifestation ; this is well illustrated in its well-established tendency to recur after extirpation of an affected lymph node. From its tendency in this direction Billroth called it "malignant lymphoma." This is also a very good name, for it commits no untruth in its meaning. The fact is often lost sight of, that the disease can with much propriety be considered a general infection of the body by a new growth, using the word in the sense that we call a cancer a new growth.

The disease attacks all ages, but is most common between the ages of ten and thirty, and rather rare in childhood ; Henry, of Philadelphia, writes that while its rarity is at any rate confessedly great, yet it is particularly so with infants and in early childhood. There appears to be a preponderance of males attacked, about 213 of the cases falling to this sex. Guiteras believes it is more common in the South than leukemia, having seen six cases in eight years. As a rule it attacks the cervical lymphatics first, and often seems to follow along the course of a great vessel, *e. g.*, along the aorta, manifesting itself in the posterior mediastinum or the retro-peritoneal lymphatics. It is not essential, however, in order to have Hodgkin's disease, that we must have all of the lymphatics of the body enlarged. Osler has reported a case where the retro-peritoneal glands alone were attacked, though the patient exhibited other symptoms of the disease ; it is also possible to have a patient suffer from splenic tumor, without any other lymphatic trouble, and yet here, as I will speak of later, it seems tenable to diagnose Hodgkin's, especially when some of the other symptoms are present. Two varieties of the lymphatic new growth have been described, the soft and the hard. This is a good distinction for gross anatomical purposes, but the distinction is based, histologically, on weak ground, for the two processes are essentially the same in each variety and both may be present in the same case. They differ only in the amount of connective tissue new growth present ; there being an excess in the hard variety, and vice versa. Such nodules as occur in pseudo-leucocythemia, as Cohnheim termed the disease under consideration, have been mistaken for tubercles when found in the lung or the pleura. There is nothing to prevent a co-existence of tuberculosis and Hodgkin's disease, and I believe that this was true in the present case, basing my opinion on what may seem insufficient ground, to-wit, the caseated bronchial lymph nodes.

The lymphatic enlargement is due to a new growth of lymphoid cells and connective tissue; the splenic growth is found infiltrating the Malpighian bodies alone, or breaking through and disseminating. A section of such an organ gives a mottled picture; the attacked Malpighian bodies appearing as firm white or yellow nodes, while the surrounding splenic tissue appears dark and normal. These growths of lymphoid matter are apt to occur where we normally find such tissue, as in Peyer's patches, in the spleen, etc., but they may be found anywhere, in the lung, brain, cord, etc., and produce symptoms varying in accordance with their sites. The minute anatomy varies slightly; in a modular new formation we may find an almost complete imitation of a true lymph node, with its trebeculæ, lymph follicle and lymph cords, etc., with, however, a little more trebecular tissue than normal. On the other hand, a non-circumscribed growth may occur, and here the tissues are merely infiltrated with lymphoid cells bound together by a delicate recticular tissue. There rarely may be giant cells present. We do not find such aggregations of cells as are peculiar to tuberculosis, and this constitutes one point of the microscopical differential diagnosis between this disease and tuberculosis. As you know, we may have a diffused tubercular tissue with no miliary tubercles, and here one might have difficulty in differentiating the two. We would here have to rely upon the presence or absence of a tendency to caseation, and the presence or absence of the characteristic bacilli.

At times a group of superficial enlarged lymphatics may inflame and suppurate, but in such a case one is usually able to trace these changes to traumatism, such as the constant irritation of clothing, etc. At best this complication is rare; some authors state that caseation can also occur; I believe that this is not true, but that in such cases a tubercular cause can always be found. The spleen in the majority of cases is enlarged, but to a very moderate degree, and as a rule we find no implication of the bone marrow. The nodal enlargement may remain in *statu quo* for a long time, may increase in size, slowly or rapidly, and may even have periods of recession. In the case now reported the lymph nodes were decidedly smaller after death, particularly in the groin and axilla. This disease has been termed diffused lymphomatosis, and if one desires he can employ the name, for histologically the term is correct. The blood is usually altered more or less, as one would anticipate by such a profound constitutional disease; moreover, this is especially to be expected if we believe that the lymphatics have anything to with the manufacture of blood. There is an apparent increase of white cells, enough at times to constitute the condition called leucocytosis, but often not more so than is observed in healthy people after a hearty meal. The reds are as a rule greatly diminished in number, and this causes an apparent increase of whites, which really are only multiplied proportionally, not absolutely. Henry, of Philadelphia, argues that this change is due to interference of the pathological changes with the manufacture incidental to the disease. The anemia, therefore, follows the lymphatic enlargement, and thus the disease cannot be classed with the so-called primary anemias. There is usually no occurrence from which the disease can be said to have started. Cases have been reported where typhoid fever, whooping cough, and particularly post-partum hemorrhage have been credited with precipitating the disease. The diagnosis of pseudo-leucocythemia lies between it and tuberculosis, carcinoma, sarcoma, and lymphoma, when the lymphatics are principally attacked, and also lymphatic leukemia; and if there be a splenic tumor, one must differentiate between Hodgkin's and malaria, leukemia, the splenic enlargements of primary anemias, typhoid, carcinoma, and echinococcus of the spleen, amyloid spleen, the splenic enlargement of hepatic cirrhosis and *anemia splenica.* Some of these differences can be more or less easily discerned, as with malaria, typhoid, echinococcus, amyloid and the tumor due to hepatic cirrhosis. At times, however, it is a matter fraught with the greatest difficulties to determine whether a given case be one of primary anemia, leucocythemia, carcinoma, Hodgkin's disease, or *anemia splenica.* Cases have been reported illustrative of every shading between the closely allied forms, leucocythemia, Hodgkin's and anemia splenica; one must here wear his best spurs, not only in order to make a correct diagnosis, and thus help his patient if possible, but also to maintain his reputation, and to advance scientific medicine and medical knowledge.

Sarcoma of the lymphatics is rather rare; it attacks any node, spreads by contiguity as a rule, and is seldom diffused throughout the body. It is said also that with both carcinomatous and sarcomatous disease of the lymphatics the nodes have a tendency to become confluent and thus not remain distinct as do the nodes of Hodgkin's; the nodes in the latter disease feel like balls freely movable under the skin, a point to which Southey called particular attention.

Tuberculosis is apt to have other manifestations of its ravages present besides an involvement of the lymphatics. We are pretty sure to find indications of it in the lungs, joints, or bones, and if a nodule be extirpated we will surely find those persistent marks of tuberculosis, miliary tubercles, caseation and, in all probability, the characteristic bacilli. The chief difficulties of a differential diagnosis are between Hodgkin's and leukemia, with a moderate amount of splenic and lymphatic enlargement.

It is as long ago as 1832 when Hodgkin first described the disease now under consideration; in 1845 Virchow gave the name "leukemia" to that malady now more generally known as leucocythemia. At that time there was not the faintest suspicion that these two diseases had anything in common, and, in fact, it has only been since the more general use and perfection of the microscope that pathologists have discovered any relation between the diseases.

In speaking of leukemia, Delafield says:

> . . . There is a form of disease in many respects, particularly in the lesion of the lymph-glands (nodes), identical with leukemia. There is, however, usually a less prominent involvement of the spleen, and what is more striking, no increase in the number of leucocytes in the blood. . . . The lesions of the lymph-glands are identical in both diseases, and it is convenient to assign different names to them simply because for reasons which we do not at all understand, they seem to arise under different conditions, and to be associated with a constant difference in the character of the blood. . . . The lesions of the spleen are essentially the same under both of these conditions.

Indeed, so close are the lines of similarity between these diseases that Langhans suggests that they be classed under the head of "lymphomatoses" with two divisions or forms,—the non-leukemic and the leukemic. Of pseudo-leucocythemia it can be said that it has three varieties: first, that form where the lymphatics are chiefly involved, or the so-called lymphatic form; second, the form where besides the lymphatics we have the spleen also involved,—in other words, a lymphatic and splenic form; and third, a pure splenic variety, in which case the spleen alone is affected. So far as I know, very few cases of the last have been reported as such. If one pauses to think, it will be realized that to recognize a pure splenic form of Hodgkin's would be no small undertaking, for one would have so few points of differentiation. There being no increase of whites with Hodgkin's, it would be hard to decide whether the enlarged spleen did not arise as a consequence of slow malarial poisoning; or, that it was not the seat of, perhaps, a malignant growth.

There are other various well-known conditions under which an enlargement of the spleen may be observed, as, for example, syphilis, obstruction to the circulation, as in hepatic cirrhosis, etc. Excluding all cases of splenic tumor unaccompanied by leucocythemia with any of the above-mentioned conditions as a cause, there yet remains a few cases where no cause can be traced. Friedrich, in 1865, described several cases of this nature. His anatomical description tallies almost exactly with that given of Hodgkin's disease.

Mosler, in *Von Ziemssen's Encyclopedia*, describes a class of cases termed hypertrophy of the spleen. These cases are of the nature of splenic enlargement, with no traceable cause, such as amyloid, syphilis, neoplastic, etc. In these cases the spleen may be diffusedly affected by the new cells, or in small spots only. Section of the organ shows it to be either hard and firm, and of a uniform whitish-yellow color, or mottled and nodular. Microscopically, these changes are seen to consist of one of two conditions, either a marked increase of lymphoid cells, most abundant in the Malpighian bodies, but at times equally common throughout the whole organ; or a striking increase of the trebecular tissue. There may be all sorts of gradations between these two. This condition of enlarged spleen, with no traceable cause, as mentioned above, is, and has been, recognized as a distinct disease. Within the past year I have had occasion to see one such case, and, in thinking over the subject, the question arose in my mind whether this change could not be considered as the pure form of Hodgkin's disease. To my satisfaction, I found, on looking over the literature touching on splenic diseases, that this same idea had been brought out long ago by several observers, particularly Banti, Strumpell, Wood, and Henry. The last-named observer, in his brochure on Anemia, draws attention in particular to this form of disease under the head of *anemia splenica*, and H. C. Wood has spoken of it as the splenic form of pseudo-leucocythemia. The reasons for considering it a form of Hodgkin's disease are, briefly: First, that the alterations are strictly similar to those found in Hodgkin's disease; and second, the marked and profound anemia which sooner or later appears. Indeed, so destructive are the blood changes that hemmorrhages are more or less frequent in this form of the disease, and one may meet with extensive losses of blood, from the gums, into the pleural cavity, etc. The microscopic changes of the blood are pronounced; the reds are notably diminished, and are of many forms, such as we find in pernicious anemia; they are sometimes enormous, comparatively speaking, in size forming the so-called poikilocyte, and the word "poikilocytosis" has been coined to designate this condition irrespective of cause; again, we often find the red cells very small, the so-called microcytes. The whites are always relatively increased; sometimes there is a moderate degree of leucocytosis, and then, again, it may almost become truly leucocythemia; the marrow is frequently the seat of changes similar to those found in cases of profound primary anemia. It is found to be darker in hue, a purplish being most common, and there is a notable loss of fat, with an increase of the cellular elements proper, particularly of the medullated red cells. In the blood itself are frequently found these same nucleated red cells; they are also altered in shape, becoming larger, and at times elongated, a condition which led Henry to term this a reversion to the repitilian type. In the so-called third stage of this disease, that of cachexia and increasing anemia, we find the same marked debility as in Hodgkin's and leukemia. I draw particular attention to *anemia splenica*, for it is here almost alone that the operation of splenectomy promises and gives benefit. Of four operated on for relief from this form of splenic disease, three recovered. Of course, in a carcinomatous or echinococcous disease of the spleen, the operation is of considerable value, but in pseudo-leukemia, leucemia, and *anemia splenica* it is in the last alone that it gives promise of being beneficial.

If we follow Langhans in his classifications we are now led to the leukemic form of diffused lymphoma, and to this I invite your attention for a few moments, to consider very briefly some of the points of similarity. As quoted above, it has been said that the majority of changes, anatomically speaking, found in leukemia, are precisely similar to those of Hodgkin's. With leukemia, however, there are more and different varieties; the pure splenic, the mixed splenic and lymphatic, and the myelogenetic forms, the latter being that form in which the disease manifests itself in the bone marrow alone; it is claimed, however, that this last variety is not tenable. With all the anatomical deviations from the normal, one must find, in order to complete the picture of leukemia, *leucocythemia*, that is to say, an alteration of the blood, as the word signifies, where the white corpuscles are markedly and absolutely increased in number. Accompanying this there is usually a more or less profound and extensive destruction of the reds, although this may not obtain in every case, for there are a number of cases reported where the reds were only slightly diminished, yet the disease progressed to a fatal termination. It is stated that the combined number of the reds and the whites does not more than equal the normal number of the reds; whether this is true or not I cannot say.

A simple increase of whites, however great, without the characteristics, splenic, lymphatic or myeloid changes, does not indicate leukemia, for Von Jaksch has found that a persistent leucocytosis of a high degree may be present in certain chronic anemias, especially in that dependent on rachitis, and it may even be present in such acute diseases as pneumonia. It is an exceedingly interesting question why there should be found in two similar diseases, anatomically speaking; in one an enormous increase of the white cells, and in the other only a slight multiplication of these same cells or no deviation at all from the normal. The greatest block to the discovery of the true relation between these diseases is the paucity of our knowledge concerning the origin of the different corpuscular elements of the blood, and their relation one to another. As one writer has put it, "We can only put forth a hypothesis, and a weak one at that." The idea which has received as much support as any is that which assumes that the red cells are made from the whites, and that the workshops where this elaboration occurs are, in order of importance, the spleen, the bone marrow, and the various lymphatic structures and collections of lymphoid tissue throughout the body, to-wit, the nodes, Peyer's patches, etc. It is further assumed that to have the transformation go on to perfection, the white cells must be detained for a somewhat protracted time in these organs, and that anything which tends to hasten the circulation through these spots will cause an unusual number of leucocytes to pass on into the circulation. The well-known post-prandial leucocytosis is explained on such grounds; there is at this time, according to this theory, a splenic hyperemia, and a consequent increased speed of circulation, with its assumed chain of hemic changes.

H. C. Wood, as long ago as 1871, made these keen observations:

Clinically, the so-called true and false leukemias are the same, save only in the matter of white corpuscles; all varieties of leukemia are represented in pseudo-leukemia. . . . Evidently there are, therefore, two dyscrasiæ which in their natural history are precisely alike; two disorders most mysterious in their causes, most complicated in their life-features, most wide-spread in their lesions, and yet preserving, as far as can be discovered in the minutest detail, absolute identity, save only in the corpuscular elements of the blood.

It has been said that a given case of Hodgkin's disease would never show a sufficient increase of whites to justify one in saying that the disease had passed into leukemia. Latterly, there have been reported some cases where careful blood counts were made daily; it is of much interest to learn that in some of these cases the leucocytes rapidly increased in number shortly before death, and one can, perhaps, explain on these observations why nearly every observer has failed to see a case of pseudo-leucocythemia which progressed to the true form.

The changes of the blood occurring in leukemia, include not only the anemia and multiplication of the white cells, but also those that affect the very nature of these white cells themselves. Ehrlich was the first to call attention to this fact, and he, together with his pupils, has done more in this line than any other worker.

It has been found that the leucocyte, in its various phases, *i. e.*, as an inflammatory cell, a connective tissue corpuscle, etc., exhibits peculiar preferences for different analine dyes. Thus Ehrlich found that all of the different varieties of leucocytes could be classed in three groups, including five varieties, which he named from alpha to epsilon; the division being based on the cell reaction when stained by acid, neutral, or basic analine colors. Those cells whose granules take or select the acid stains are termed the (1) eosinophiles: they are colored deeply by eosin particularly, but also by acid fuchsin, aurantia, etc. Under the second division Ehrlich places all those leucocytes, or their modifications, whose granules are most intensely colored by basic analine pigments; these are termed (2) basophiles. Gentian-violet, methyl-violet, methyl-green, etc., are incorporated by these cells with great avidity, and the cell granules are correspondingly stained. The third class includes those cells whose protoplasmic granules are most highly colored by such neutral analine dyes as methyl-blue and acid-fuchsin combined, or by picrate of rosanaline, and to these the name of (3) neutrophiles is applied.

In regard to the significance of these facts it can be said that the basophile cell is found but rarely in the blood; it is more common in connective tissue as the so-called "mastzellen" and has but little interest for the diagnostician. The neutrophile cell is the leucocyte common to the blood in health and leucocytosis; while the eosinophile is only of rare occurrence in the blood as a normal constituent. It is, however, greatly increased in leucemia and slightly so in some other diseases.

The importance of these changes in the white cell can easily, on a moment's thought, be seen to be of great importance; if one has a case of slight general lymphatic enlargement, or splenic tumor, with leucocytosis, and on examination of the blood it be found that there is a large number of eosinophiles present, then one is at once warned to be guarded in giving a prognosis, for the abundant eosinophiles stamp the malady at once, even though it be in its veriest inception, as leukemia, and not simple adenopathy consequent in anemia, syphilis, etc. Such a condition of the blood would also enable one to differentiate between Hodgkin's and leucėmia.

Besides these chromogenic peculiarities, which are held to be correct and diagnostic by so thorough and careful a worker as V. Jaksch, there are also said to be differences in the morphology of the white corpuscles in the various varieties of leucemia. On this point V. Jaksch writes:

> When large and small leucocytės—the latter predominating—are found in the blood, the leucemia is of a lymphatico-splenic character. When, on the other hand, the large cells alone are found, we are justified in concluding that the disease is splenic, with little involvement of the lymphatic glands or marrow. If many corpuscles of a transitional form are found, nucleated red cells, and especially large white multinuclear corpuscles, there remains no doubt that the bone marrow is the seat of serious changes, and the disease is of the myelogenic type.

The diseases which we have so briefly reviewed, form a remarkable group, and shade one into another with striking smoothness. While the points of distinction are of paramount interest—in one to the surgeon, indicating to him the utility or futility of removing a group of enlarged lymphatics or an enlarged spleen; in the other demonstrating to the physician the uselessness of quinine as a therapeutic measure, and in the third giving both the surgeon and the physician a ray of hope in the possibilities of splenectomy—yet to us as members of a pathological society, it seems to me to be more interesting, and to be a deeper penetration into territory seldom visited by the average physician, to think for a moment on points concerning the ultimate causes of these maladies. Of course, it is needless to remind you that we are now just as densely ignorant of the first causes of these diseases as we are of those of carcinoma or sarcoma. Westphal believes that leukemia is a specific infectious disease, although all his attempts to transmit it to animals have proven abortive. It is the fashion of the day to draw at sight on bacteria, and to hope that in some member of this great vegetable group will be found the essential cause of all such diseases as have heretofore baffled our efforts at a solution of their causes. Attention is now being drawn to the possibility of lowly organized animal forms, the protozoa, etc., acting as causes of disease, and such a relation will probably have to be sought for in those diseases as have failed to exhibit peculiar bacterial relations. We all recall the association of the plasmodium with malaria; the *filaria* with certain types of elephantiasis, the *ameba coli* with some varieties of dysentery and hepatic abscesses. Characteristic *coccidia* have been found in *molluscum contagiosum* by Pfeiffer and Neisser; in epithelioma by Malassez, and in Paget's disease. All of these are examples of the association of animal forms with disease, though the exact relations in these cases are hard to define, because of our insufficient knowledge. So far as I know, all attempts to cultivate these creatures have failed, and in this we are at once heavily handicapped. Koch, in his recent address before the International Congress, said that he had hoped and believed that these organisms would soon be artificially cultivated. It is hard to imagine what prevents an animal from developing Hodgkin's disease, if the affection be infectious, when some of the blood, or the lymphoid matter, is injected into the circulation of such an animal. And yet so much depends on what at first may seem trivial differences, that one must be exceedingly cautious if he tend to draw conclusions from repeated failures to transmit a given, supposed infectious disease. For example, it was long well known that chickens were peculiarly resistant to anthrax, and that a hen could withstand and, in fact, could not be infected with a relatively enormous dose of a pure culture of the bacillus anthracis; and until Pasteur proved that this was due to the fowl's normal high temperature, it was used as a fact that was said to controvert the truth of the ultimate relation which we now know exists between charbon and the bacillus of anthrax.

I do not believe it is at all visionary to hope that the coming few years will, perhaps, reveal protozoa or other animal form, as causes of the diseases now under consideration. These animal forms are commoner in the warmer temperate and torrid zones, being, perhaps, not rugged enough to withstand our colder climate.

This is true of the filaria, both of that form found in man and that found in birds. Sutton states that if a number of negroes, in some regions of torrid Africa, be taken at random and their blood be examined, that fully fifty per cent. will show filaria, even though but a very small per cent. may exhibit clinical evidences of disease. Guiteras, as mentioned above, says that Hodgkin's disease is more common in the South than leukemia, and, in fact, the number of cases which he met seems to show that the disease is itself more frequent South than North. If this be true, though no claim to this effect is made, it would be a mild parallelism to the greater prevalence of malaria, elephantiasis, etc., in southern regions, these two diseases being exampled because of their supposed animal origin. It is not throwing too great a burden on some animal form to ascribe to it the changes found in leucocythemia; true, there are here great and apparent gross lesions present, but equally marked and manifest anatomical alterations are to be seen in both of the diseases just mentioned above, and they are admittedly of animal origin.

In reviewing the subject even as hastily and incompletely as I have, one thing impresses me with renewed force, viz., that with the instruments of precision which we now have at our command, we ought to spare no efforts in our investigations of such cases as we have spoken of tonight, and I feel that if the average worker would study his cases more closely much of great importance and good would result.

